# Exploring the therapeutic potential of precision T-Cell Receptors (TCRs) in targeting KRAS G12D cancer through *in vitro* development

**DOI:** 10.32604/or.2024.056565

**Published:** 2024-11-13

**Authors:** WEITAO ZHENG, DONG JIANG, SONGEN CHEN, MEILING WU, BAOQI YAN, JIAHUI ZHAI, YUNQIANG SHI, BIN XIE, XINGWANG XIE, KANGHONG HU, WENXUE MA

**Affiliations:** 1 Sino-German Biomedical Center, National “111” Center for Cellular Regulation and Molecular Pharmaceutics, Cooperative Innovation Center of Industrial Fermentation (Ministry of Education of China & Hubei Province), Hubei University of Technology, Wuhan, 430068, China; 2 Center of Research & Development, Beijing CorreGene Biotechnology Co., Ltd., Beijing, 102206, China; 3 Department of Medicine, Sanford Stem Cell Institute and Moores Cancer Center, University of California San Diego, La Jolla, CA 92093, USA

**Keywords:** T cell receptor (TCR), TCR therapy, Tumor-infiltrating lymphocytes (TILs), Kirsten rat sarcoma virus (KRAS), G12D, Alloreactivity

## Abstract

**Objectives:**

The Kirsten rat sarcoma virus (KRAS) G12D oncogenic mutation poses a significant challenge in treating solid tumors due to the lack of specific and effective therapeutic interventions. This study aims to explore innovative approaches in T cell receptor (TCR) engineering and characterization to target the KRAS G12D_7-16_ mutation, providing potential strategies for overcoming this therapeutic challenge.

**Methods:**

In this innovative study, we engineered and characterized two T cell receptors (TCRs), KDA11-01 and KDA11-02 with high affinity for the KRAS G12D_7-16_ mutation. These TCRs were isolated from tumor-infiltrating lymphocytes (TILs) derived from tumor tissues of patients with the KRAS G12D mutation. We assessed their specificity and anti-tumor activity *in vitro* using various cancer cell lines.

**Results:**

KDA11-01 and KDA11-02 demonstrated exceptional specificity for the HLA-A*11:01-restricted KRAS G12D_7-16_ epitope, significantly inducing IFN-γ release and eliminating tumor cells without cross-reactivity or alloreactivity.

**Conclusions:**

The successful development of KDA11-01 and KDA11-02 introduces a novel and precise TCR-based therapeutic strategy against KRAS G12D mutation, showing potential for significant advancements in cancer immunotherapy.

**Highlights**

- We developed two high-affinity TCRs, KDA11-01 and KDA11-02, specifically targeting the KRAS G12D mutation to enhance precision.

- The TCRs demonstrated precise recognition and elimination of tumor cells with the KRAS G12D mutation, advancing targeted therapies.
- The TCRs exhibited strong *in vitro* anti-tumor effects, including significant IFN-γ production and cytotoxicity, with no cross-reactivity observed.
- Comprehensive testing confirmed minimal alloreactivity, supporting the safety and specificity of these TCRs for therapeutic applications.
- This study highlights the potential of TCR-based therapies for targeting tumors with specific mutations, providing new opportunities in personalized cancer immunotherapy.

## Introduction

The oncogenic Rat sarcoma virus (RAS) oncogene family, including Kirsten rat sarcoma virus (KRAS), Neuroblastoma RAS viral oncogene homolog (NARS), and Harvey rat sarcoma virus oncogene homolog (HRAS) mutations, represents a significant subset of genetic aberrations in human epithelial cancers, occurring in approximately 20% of cases [1]. Among these, kras mutations are the most prevalent, accounting for 86% of ras-associated oncogenic mutations. These mutations are particularly common in pancreatic cancer (45%), colorectal cancer (crc, 13%), and lung cancer (4%) [2–4]. Notably, kras codon 12 mutations, especially the g12d variant, have emerged as critical therapeutic targets due to their high frequency and oncogenic effects [5,6].

Targeting KRAS mutations has been historically challenging due to the unique structural properties of KRAS, which have made it resistant to small-molecule inhibition. Most clinical efforts have focused on the G12C variant, often associated with lung cancer [7,8]. While these efforts have led to the development of targeted therapies, such as the FDA-approved G12C inhibitor AMG510 (Sotorasib), they have been limited by drug resistance and poor prognosis, particularly for KRAS G12C mutation carriers [9,10]. Recently, Adagrasib, another targeted inhibitor, has been approved, expanding treatment options for KRAS G12C mutations. However, there remains a significant lack of effective treatments for KRAS G12D and G12V mutations, which are particularly relevant in pancreatic cancer and CRC [7,8]. MRTX1133, a potent and selective inhibitor of KRAS G12D, is currently undergoing clinical trials, further underscoring the need for diverse therapeutic strategies targeting these mutations [11,12].

T-cell receptor (TCR)-based immunotherapy has emerged as a promising approach, leveraging the tumor-specific and immunogenic nature of KRAS mutations. Early clinical studies have highlighted the potential of TCR-engineered T cells targeting KRAS G12D mutations to induce tumor regression in metastatic cases [13,14]. However, the clinical utility of these therapies is often limited by the necessity for human leukocyte antigen (HLA) compatibility, as HLA alleles such as HLA-C*08:02 and HLA-A*11:01 vary significantly across different ethnic groups. This variation affects the broader applicability of these therapies across diverse populations [15,16]. Therefore, this limitation underscores the need for a broader repertoire of TCRs that can target KRAS mutations across various HLA types, thereby enhancing the clinical utility and accessibility of TCR-based therapies.

In this study, we aimed to address these challenges by employing TCR αβ single-chain technology to isolate and engineer TCRs from human tumor-infiltrating lymphocytes (TILs). We utilized an innovative fusion PCR technique to link the separately amplified TCRα and β chains, which allowed us to construct comprehensive TCR sequences without the need for the complex equipment or procedures previously reported [17,18]. This methodology improves the efficiency of TCR assembly, enabling the direct use of single-cell amplified products for subsequent fusion PCR, and leading to a cost-effective and rapid assembly of a complete TCR sequence.

The practicality and safety of these TCRs were validated *in vitro* through various assays assessing affinity, activation, proliferation, cytotoxicity, and alloreactivity after expansion and culture. By adding more TCRs specific to KRAS G12D in the context of HLA-A*11:01, our findings address the compatibility concern associated with existing therapies and broaden the scope of TCR therapy within oncology. This study contributes to the ongoing development of cancer therapeutics by providing potent treatment alternatives that can be applied to a wider patient population, representing a significant advancement in precision TCR-based immunotherapy.

## Materials and Methods

### Tissue specimen collection and ethical approval

This study, involving human specimens, adhered strictly to the ethical guidelines set by the Hubei University of Technology, aligning with the principles of the 1964 Helsinki Declaration and its subsequent updates. Ethical clearance was granted by the Life Science Ethical Review Committee at the Sino-German Biomedical Center, Hubei University of Technology as of 1 July 2021. With written informed consent, tumor tissues (ranging from 0.2 to 2 cm^3^ in volume) and 10 mL peripheral blood mononuclear cells (PBMCs) were obtained from seven patients during surgical resection. The inclusion criteria for the study encompassed solid tumors such as colorectal cancer (CRC), intrahepatic cholangiocarcinoma (IHC), pancreatic cancer, and lung cancer. This study focuses on two specific patients: a female with CRC (Patient ID: LYL210724) and a male with intrahepatic cholangiocarcinoma (ICC, Patient ID: WFY210825).

### Patient selection and sample collection

Tumor tissue samples were obtained from two patients diagnosed with CRC, ICC respectively at the Affiliated Zhongnan Hospital of Wuhan University, China. Patient selection was based on the availability of sufficient tumor tissue and specific HLA type. All patients provided informed consent in accordance with our IRB/Ethical approval # 202107-19231 from the Ethics Review Committee of Life Sciences, Sino-German Biomedical Center, Hubei University of Technology. The tumor tissues were processed as described to isolate T cells for TCR analysis and selection.

### Cell lines and reagents

We utilized various human cancer cell lines including PANC-1 (pancreatic, HLA-A*11:01+, KRAS G12D+), HPAF-II (pancreatic, HLA-A*11:01-, KRAS G12D+), HeLa (cervical, HLA-A*11:01+, KRAS G12D+), LS180 (colon, HLA-A*11:01-, KRAS G12D+), and HuCCT1 (cholangiocarcinoma, HLA-A*11:01+, KRAS G12D+), sourced from the Cell Bank of Chinese Academy of Sciences Shanghai. All cell lines were regularly tested to confirm they were mycoplasma negative. We generated the 63 different HLA class I variant cell lines in-house. The T2 cell line, with a compromised ability to present endogenous antigens, and the 293 T cell line (CRL-3216 | ATCC), were purchased from the American Type Culture Collection (ATCC, Manassas, VA, USA). These cells were genetically modified to lack HLA genes and express HLA-A*11:01, serving as antigen-presenting cells (T2KO-A1101).

All the cell lines were cultured in their recommended basal media IMDM for T2 and T2KO-A T2KO-LUC.2-TAP1101, DMEM or RPMI-1640 for others supplemented with 10% fetal bovine serum (FBS, v/v, GeminiBio, West Sacramento, CA, USA) and 1% penicillin/streptomycin (v/v, Gibco, Thermo Fisher Scientific, Waltham, MA, USA) in a 37°C humidified incubator with 5% CO_2_, as per ATCC guidelines.

Synthetic peptides corresponding to KRAS mutations G12D (KD2, KD1), G12V (KV2, KV1), G12C (KC2, KC1), and wild-type KRAS (KWT2, KWT1), with >95% purity, were obtained from GenScript Biotech, Nanjing, China. Tetramers were procured from MBL Beijing Biotech. Peptides were dissolved in DMSO to a concentration of 10 mM, aliquoted, and stored at −80°C.

Jurkat E6.1 cells, also from ATCC, were modified to express CD8 coreceptors and an eGFP/CD69 reporter driven by the nuclear factor of activated T cells (NFAT), with their endogenous TCRs knocked out, to create a reporter cell line.

### Generation of different HLA class I variant cell lines

To generate a panel of HLA class I variant cell lines, we used the T2-KO-LUC.2-TAP cell line, which has its endogenous HLA genes knocked out. Initially, the cells were checked for viability, centrifuged, at 1250 rpm/minute for 5 min at 4°C, and then adjusted to a density of 1 × 10^6^ cells/mL. For transduction, 1 mL of the cell suspension was added to each well of a 6-well plate, and lentiviral vectors were introduced based on a multiplicity of infection (MOI) of 10. Without viral titer data, varying volumes of the virus (5, 10, 20, 50, and 100 µL) were employed to transduce 1 × 10^6^ cells, followed by overnight incubation to facilitate viral entry.

The next day, the medium was refreshed. 48 h post-transduction, selection commenced using Puromycin at a final concentration of 1 µg/mL, continuing for one week to ensure complete elimination of cells in the negative control wells, thereby confirming successful transduction and resistance to Puromycin.

Before freezing and storing the cell lines, it is crucial to perform phenotypic testing to confirm HLA expression. This verification is conducted through flow cytometry analysis using the BD FACSAria™ III (BD Biosciences, San Jose, CA, USA) to ensure accurate HLA profiling.

### TIL generation, expansion, and KRAS G12D-specific T cell selection

Postoperative fresh tumor specimens were processed immediately, undergoing a sterile saline wash before being sectioned into 2 mm fragments for TIL culture in 6-well plates. The culture medium consisted of 90% X-VIVO15 (Lonza, Suzhou, China) enriched with 10% human AB serum (GemCell™, Sydney, NSW, Australia) and IL-2 at a concentration of 12000 IU/mL (ShuangLu Ltd., Beijing, China). On day two, expansion was facilitated by the addition of anti-CD3/CD28 Dynabeads^®^ (Thermo Fisher, MA, USA), with half of the medium and IL-2 refreshed bi-daily from the third day. Before sorting, T lymphocytes were characterized for CD3, CD4, and CD8 expression. After 14–15 days of culturing, CD3^+^CD8^+^ cells positive for the MHC tetramer specific to HLA-A*11:01/KD2 were isolated via single-cell flow cytometry sorting into 96-well plates.

**Figure 1:**
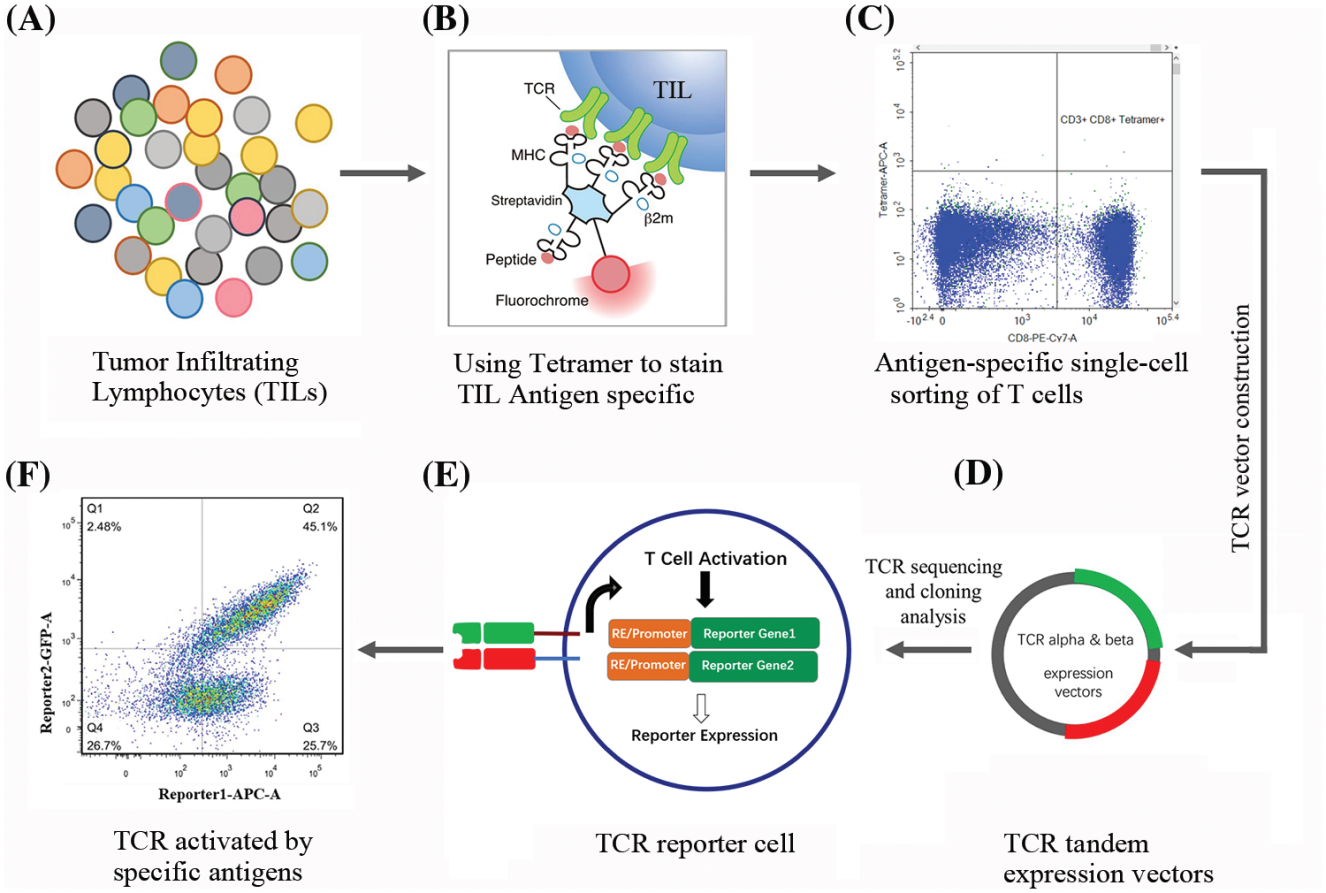
Workflow for isolation, identification, and characterization of TIL derived TCRs. (A) illustrates the diversity of TILs within the tumor microenvironment (TME), with different colors representing distinct T cell subsets. (B) details the tetramer staining process utilized to identify TILs with specific antigen recognition. Here, tetramers bind to the TCRs on TILs that specifically recognize the presented peptide antigen. (C) shows the gating strategy used to isolate antigen-specific T cells. Cells that are positive for both CD8 and the tetramer are identified as specific to the target antigen. (D) presents a schematic of the TCR vector construction process. TCR α and β chains are linked to form tandem expression vectors for subsequent transduction into recipient cells. The plasmids were sequenced to confirm the specific TCR sequences. (E) illustrates the TCR reporter cell, highlighting the integration of the specific TCR along with a reporter gene construct. The reporter genes are activated upon successful TCR-mediated cell activation following antigen recognition. (F) demonstrates flow cytometry analysis showing a shift in fluorescence intensity, indicating the expression of reporter genes triggered by antigen-specific TCR activation.

Fig. 1 provides a schematic overview of the TIL isolation, expansion, and subsequent selection of antigen-specific T cells, offering a visual representation of the key steps described.

### Amplification of TCR αβ single-chain sequences

The TCR gene sequences were determined from flow-sorted single TIL cells. After cell lysis, cDNA was synthesized using a specific primer set for 5′-RACE PCR. Subsequent PCR amplification of the TCR α and β chains was performed using the cDNA as a template. For the TCR β-chain amplification, the first T2A peptide sequence (EGRGSLLTCGDVEENPGP from Thosea Asigna Virus) was incorporated at the 3′ end of the TCRβC region. For the α-chain, a third T2A sequence was appended to the 5′ end of the TCRαV region. This single-stranded α and β sequences underwent overlapping PCR [19] to construct the TRBV-TRBC-T2A-TRAV-TRAC (partial) amplicon, which was then cloned into the lentiCas9-Blast vector (Plasmid#52962, Addgene, Beijing, China).

**Figure 2:**
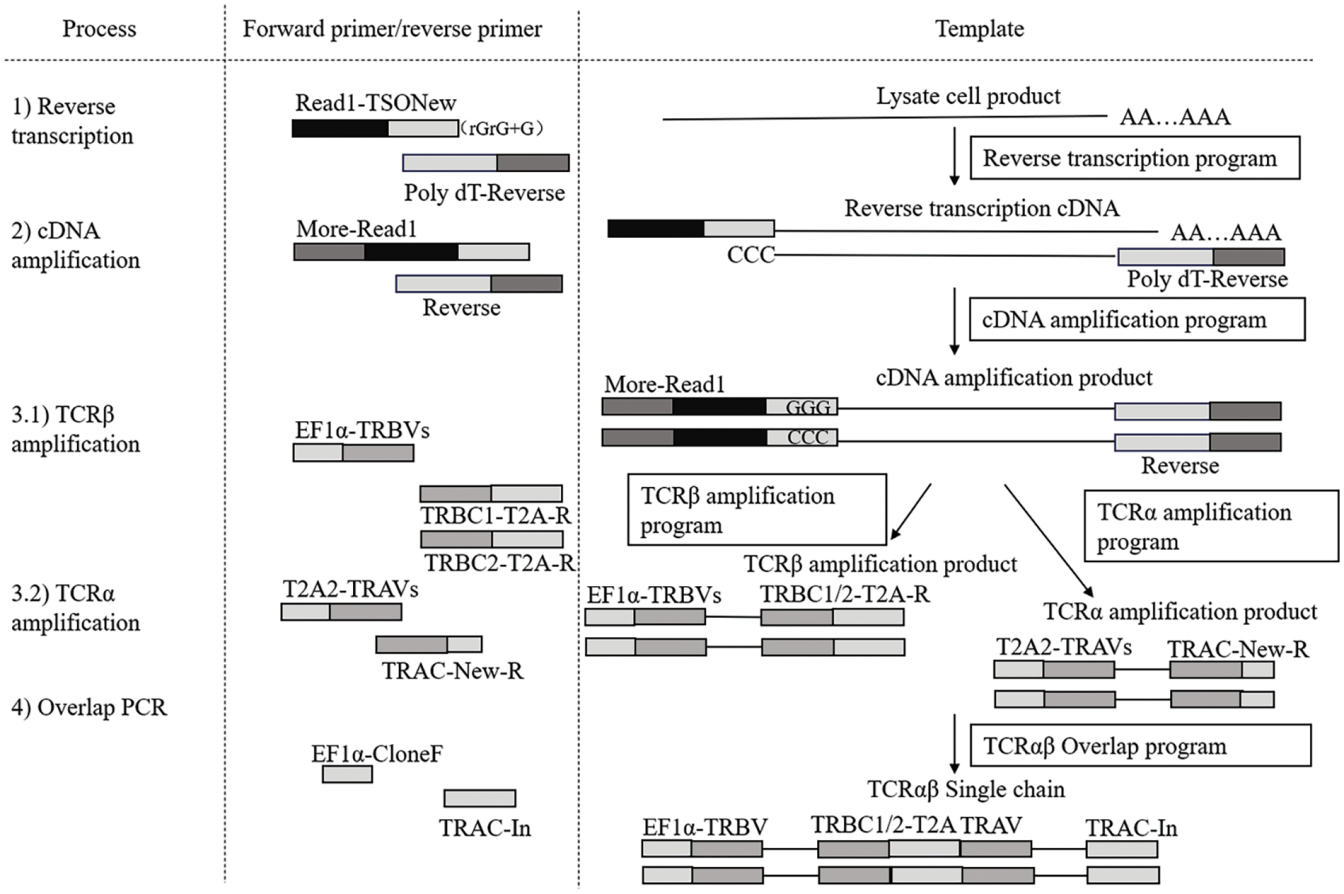
Schematic of single-cell TCR cloning procedures. This flowchart outlines the TCR cloning process, including reverse transcription, cDNA amplification, TCRα and TCRβ chain amplification, and overlap PCR. The diagram shows the sequential steps, primers used, and templates involved, starting from cell lysate to the final construction of TCRα and TCRβ single chains.

To visualize the sequential steps involved in TCR cloning, including reverse transcription, cDNA amplification, and overlap PCR, Fig. 2 presents a schematic flowchart that outlines the entire process in three columns. The first column lists the cloning process steps sequentially: 1) Reverse transcription, 2) cDNA amplification, 3.1) TCRβ chain amplification, 3.2) TCRα chain amplification, and 4) overlap PCR. The second column specifies the primers used in each step (forward and reverse primers), while the third column describes the templates used at each stage, beginning with lysate cell products for reverse transcription to generate cDNA and culminating in the construction of TCRα and TCRβ single chains.

The primer sequences used were as follows: for reverse transcription, the forward primer Read1-TSO New was 5′-CTACACGACGCTCTTCCGATCTTTTCTTATT/rGrG/+G-3′, and the reverse transcriptase primer Poly dT-Reverse was 5′-AAGCAGTGGTATCAACGCAGAGTACT[PolyT]-TVN-3′. The β-chain amplification employed the forward primer set EF1α-TRBVs [20], with the reverse set TRBC1/2-T2A-R. For the α-chain, the forward primer set was T2A2-TRAVs, with the reverse set TRAV-New-R. Finally, the overlapping PCR utilized the forward primer EF1α-Clone F: 5′-GGTTTGCCGCCAGAACAGAACACAGGACC-3′ and the reverse primer TRAC-In: 5′-AGTCTCTCAGCTGGTACACG-3′.

### Lentiviral vector production

293 T cells were cultured to reach 1 × 10^6^ per well in a 6-well plate. They were transfected with 2.4 μg of TCR encoding plasmids, 1.2 μg pspax 2 (Plasmid#12260), and 0.8 μg of vsv. g (Plasmid#14888, Addgene, Watertown, MA, USA), using the PEI MAX (MW 40000) (Polysciences, Warrington, PA, USA). The medium, DMEM supplemented with 10% FBS, was refreshed after 18 h. Viral supernatants were harvested at 48- and 72-h post-transfection.

### Verification of TCR functionality

TCRs were introduced into Jurkat reporter cells. Co-incubation with T2KO-A1101 cells loaded with the KRASG12D peptide followed. Reporter cell activation was assessed overnight using flow cytometry to detect reporter gene expression. Successfully activated TCRs were sequenced using Sanger ABI 3730 Sequencer (Thermo Fisher Scientific, Waltham, MA, USA) to confirm their specific sequences.

### Generation of KDA11-01/02 TCR-modified T cells

PBMCs from healthy donors were obtained via Ficoll-Hypaque gradient centrifugation using (Merck KGaA, Darmstadt, Germany), adjusted to a concentration of 1 × 10^7^ cells/mL, and cryopreserved. The PBMCs were sourced from three Chinese male donors aged 26–32 years old. Pre-transduction treatment included activation with anti-CD3/CD28 Dynabeads^®^ and 100 U/mL IL-2. On the second day, PBMCs were transduced with lentiviral vectors carrying the TCR genes at a multiplicity of infection (MOI) of 30. Media and IL-2 replenishment occurred every 2–3 days post-transduction to promote expansion, and on day 10, cells were collected. TCR expression was assessed by flow cytometry using anti-CD3-FITC, anti-CD4-PE, anti-CD8-PE-Cy7, and anti-mouse TCRβ antibodies. The TCR-engineered T cells were either prepared for subsequent experiments or stored for future use.

### TCR avidity evaluation

To determine TCR functional avidity, we conducted an assay using titrated concentrations of peptides presented by T2KO-A1101 cells to engage TCR-transduced T cells overnight at 37°C. We employed an IFN-γ release assay, with T2KO-A1101 cells pulsed with varying concentrations of the HLA-A*11:01-restricted KRAS G12D_7–16_ antigen peptide (KD2, ranging from 10^−4^ M to 10^−10^ M) as targets. The cells were plated at 1 × 10^6^ cells/mL in 24-well plates and cultured in RPMI-1640 medium supplemented with 10% FBS, 1% penicillin/streptomycin, and 2 mM L-glutamine at 37°C in a 5% CO_2_ incubator. The IFN-γ levels in the supernatants were measured after overnight coculture. Data were analyzed to ascertain the half-maximal effective concentration (EC50) of the TCRs, utilizing GraphPad Prism 8.0. This process was replicated thrice, each with technical repeats.

### Fluorescence-activated cell sorting (FACS) analyses

For TIL identification and sorting, antibodies such as CD3-FITC, CD4-PE and CD8-PE Cy7 (all at 1:20 dilution, BioLegend, PerkinElmer Inc., San Diego, CA, USA), along with APC-tetramer specific for HLA-A*11:01 KRAS G12D tetramer (peptide sequence VVVGADGVGK, MBL; 1:20 dilution) were utilized. TCR-modified peripheral blood lymphocytes (PBL) were stained with anti-CD3, -CD8, and -mouse TCRβ chain constant region antibodies (1:50 dilution, BioLegend, PerkinElmer Inc., CA, USA), followed by FACS analysis. Live cell verification was performed using 7-AAD staining [21] (1:100 dilution; BD Biosciences, NJ, USA), and the results were analyzed with FlowJo7.6.1 software (Tree Star, Ashland, OR, USA) focusing on live (7-AAD negative) CD3^+^ T cells.

### Cytotoxicity assay

A luciferase-based assay was employed to evaluate the cytolytic activity of TCR-transduced T cells [22]. Each well of a 96-well black plate contained 1 × 10^5^ eGFP/Fluc expressing tumor cells (PANC-1, HeLa, LS180, HPAF-II, HuCCT1) or peptide-loaded T2KO-A1101 cells, which were co-incubated with either TCR-transduced or non-transduced PBMCs in 200 μL of complete medium, in triplicate. T2KO-A1101 cells, prepared at 1 × 10^6^/mL in IMDM medium, were pulsed with 10^−6^ M antigen peptides (KC1, KC2, KV1, KV2, KWT1, KWT2, KD1 and KD2) at 37°C for 2 h for peptide loading. After a 16–24 h incubation period of the TCR-transduced T cells with target cells, 100 μL of supernatant was carefully removed. Subsequently, luciferase substrate was added to each well and incubated for 10 min. The resulting bioluminescence was then quantitatively measured using a BioTek Synergy plate reader (Thermo Fisher Scientific, MA, USA). The assay included control groups, including non-transduced PBMCs and non-target cells, to validate the specificity and effectiveness of the TCR-transduced T cells. The experiment was repeated 3 times, with technical replications. Cytotoxicity was quantified by measuring the release of tumor-associated antigens, as calculated by the formula provided below [23]:

$$\begin{array}{rl} & Cytotoxicity\;\%=\\ & \left(1-\frac{bioluminescent\;unit\;of\;experiment\;well}{bioluminescent\;unit\;of\;target\;cells\;only\;well}\right)\times 100 \end{array}$$

### ELISA for IFN- γ detection and ALLO-reactivity testing

To assess the production of IFN-γ and analyze alloreactivity, TCR-transduced and non-transduced T-cells were co-cultured with either eGFP/Fluc-expressing tumor cells or peptide-loaded T2KO-A1101 cells. For the setup, 1 × 10^5^ cells were placed in each well of 96-well black plates in triplicate, within 200 μL of the complete medium. T2KO-A1101 cells were washed twice with 1 × PBS, resuspended in IMDM medium without FBS at a density of 1 × 10^6^ cells/mL, and pulsed with the KRAS G12D_7−16_ peptide at a concentration of 10^−6^ M for 2 h at 37°C. Subsequently, FBS (10% heat-inactivated) was added to the cells. After 24 h of co-culturing, supernatants were collected and the IFN-γ levels were quantified via Enzyme-Linked Immune Sorbent Assay (ELISA), following manufacturer’s protocols, with readings taken on a BioTek Synergy plate reader (Thermo Fisher Scientific, MA, USA).

For alloreactivity analysis, T2KO- LUC.2-TAP1 cells (T2 cells with HLA knockout and restored TAP1 expression for peptide presentation) were used, which cover over 85% of the population’s HLA types [16,24]. A panel of 63 different HLA cell lines served as target cells, while KDA11-01/02 TCR-transduced T cells acted as effector cells. Both target cells and effector cells were co-cultured overnight at a concentration of 1 × 10^5^ cells/mL in RPMI-1640 supplemented with 10% FBS, 1% penicillin/streptomycin, and 2 mM L-glutamine at 37°C in a 5% CO_2_ incubator, and IFN-γ secretion in the supernatant was detected using an ELISA Kit (Cat# EH008-96, ExCell Bio Group, Suzhou, China). T2KO-A1101 loaded with 10^−6^ M KD2 peptide and PBMC served as a control, and the corresponding effector cells of PBMC at the same concentration were used as controls. The entire process was replicated three times for robustness.

### Enzyme-linked immunospot (ELISpot) assay

To confirm the functionality of TCR-transduced T cells, ELISpot assays were conducted to measure IFN-γ production. This assay evaluates the capacity of the TCR-transduced T cells, specifically KDA11-01 and KDA11-02, to recognize and respond to the KRAS G12D epitope presented by target T2KO-A*11:01 cells that were pulsed with specific peptides (KC1: VVGACGVGK, KC2: VVVGACGVGK, KD1: VVGADGVGK, KD2: VVVGADGVGK, KV1: VVGAVGVGK, KV2: VVVGAVGVGK, KWT1: VVGAGGVGK, and KWT2: VVVGAGGVGK) at a concentration of 10^−6^ M. Following the protocol outlined in our previous publications [25], ELISpot plates were coated with anti-IFN-γ antibody and blocked with FBS. The effector TCR-transduced T cells and target cells were co-cultured in a 1:1 ratio, with each type contributing 50,000 cells, in RPMI-1640 supplemented with 10% FBS, 1% penicillin/streptomycin, and 2 mM L-glutamine at 37°C in a 5% CO_2_ incubator. IFN-γ production was detected using biotinylated secondary antibodies (Mabtech, Cat #3420-2HST-10), an avidin-HRP complex (Mabtech, Cat # MABT-340), and AEC substrate (Mabtech, Cat # MABT-140). The response was quantified with a Mabtech IRIS FluoroSpot/ELISpot reader. The assay, performed in triplicate, assesses IFN-γ spot formation to directly indicate T-cell activation. Control wells containing PBMCs served to establish a baseline for background cytokine production, facilitating an accurate evaluation of the immune response elicited by the transduced TCRs.

### In vitro T cells proliferation assay

KDA11-01/02 TCR-transduced T cells and non-transduced PBMCs were preconditioned in X VIVO-15 without IL-2 for 24 h. They were then labeled with 5 mΜ CFSE [5-(and 6)-carboxyfluorescein diacetate succinimidyl ester] (Invitrogen, Thermo Fisher, MA, USA) for 5 min at 37°C with agitation. The CFSE labeling efficiency was assessed by flow cytometry immediately after labeling, with 100% of cells showing successful CFSE incorporation. After two washes in a complete RPMI medium, cells were co-cultured with KRAS G12D_7–16_ peptide-pulsed T2KO-A1101 cells at a density of 1 × 10^6^ cells/well into 24-well plates with 1mL medium. The KRAS G12D_7-16_ peptide, with the sequence VVVGADGVGK was used at a concentration of 10^−6^ M. After 5 days of co-culture, the mixed cells were stained with mouse TCRβ-APC and human CD8-PE Cy7 mAbs for proliferation analysis. T2KO-A1101 cells pulsed with irrelevant peptides served as peptide controls and PBMCs without TCR-transduction were used as the negative control. This procedure was performed in triplicate.

### Statistical methods

Data were analyzed using GraphPad Prism 8.0 software (GraphPad, San Diego, CA, USA). The student’s *t*-test and repeated measures ANOVA were employed for statistical evaluation. Histograms and line charts display the standard error of the median for each experimental group. Bidirectional tests were applied, with a *p*-value of <0.05 indicating statistical significance. Biological replicates for all experiments numbered at least three.

## Results

### Identification of KRAS G12D7-16 specific TCR, KDA11-01/02, Restricted by HLA-A*11:01

In this study, we successfully isolated TILs from a CRC specimen of a female patient (LYL210724) and an ICC specimen from a male patient (WFY210825) with both KRAS G12D mutation and HLA-A*11:01 positive. Through *in vitro* expansion and specific antigen recognition via tetramer staining, we sorted for tetramer-positive T cells. The isolated TCRs were then amplified and cloned into lentiviral vectors for further characterization.

Utilizing a Jurkat reporter cell line with knocked-out endogenous TCR, we introduced these TCRs to assess their reactivity to the target antigen presented by HLA-A11:01. The successful activation of these cells, as evidenced by NFAT-driven gene expression detected by FACS, led to the identification of two specific TCRs, KDA11-01 and KDA11-02, targeting the G12D-HLAA11:01 epitope.

From the LYL210724 sample, 9 strains of HLA*A11:01+KD2 tetramer+ single cells were isolated. After PCR amplification, overlapping PCR, and sequencing, 22 positive clones were identified, resulting in the construction of 20 TCRs in lentiviral vectors. Subsequent transfection into the reporter cell line and verification confirmed that the TCR designated as KDA11-01 was successfully activated.

Similarly, from the WFY210825 sample, 8 cell lines were isolated, leading to the identification of 38 TCRB-TCRA pairs after PCR. After recombinant identification and resistance screening, one TCR was found to be functionally active. This TCR, designated as KDA11-02, was confirmed after three rounds of activation verification.

This resulted in the identification of two TCRs specific for the G12D-HLA*A11:01 epitope, designated as KDA11-01 and KDA11-02. Detailed characteristics of these TCRs, reactive to HLA-A*11:01-KD2, are summarized in Table 1 below.

**Table 1:** Comparison of TCR features between KDA11-01 and KDA11-02

TCR	KDA11-01	KDA11-02
HLA	HLA-A*11:01	HLA-A*11:01
KRAS G12D specific	VVVGADGVGK	VVVGADGVGK
TCR Vα	TRAV12-2*01/TRAJ17*01	TRAV12-1*01/TRAJ31*01
TCR Vβ	TRBV11-2*03/TRBJ1-6*02	TRBV27*01/TRBJ-1*01

To circumvent potential mismatches with endogenous TCR sequences in human T cells, the α and β chains of the TCRs were linked to murine constant regions and arranged in a β-2A-α configuration within lentiviral vectors (Fig. 2).

### The avidity and membrane stability of KDA11-01/02 TCR in transduced T cells

**Figure 3:**
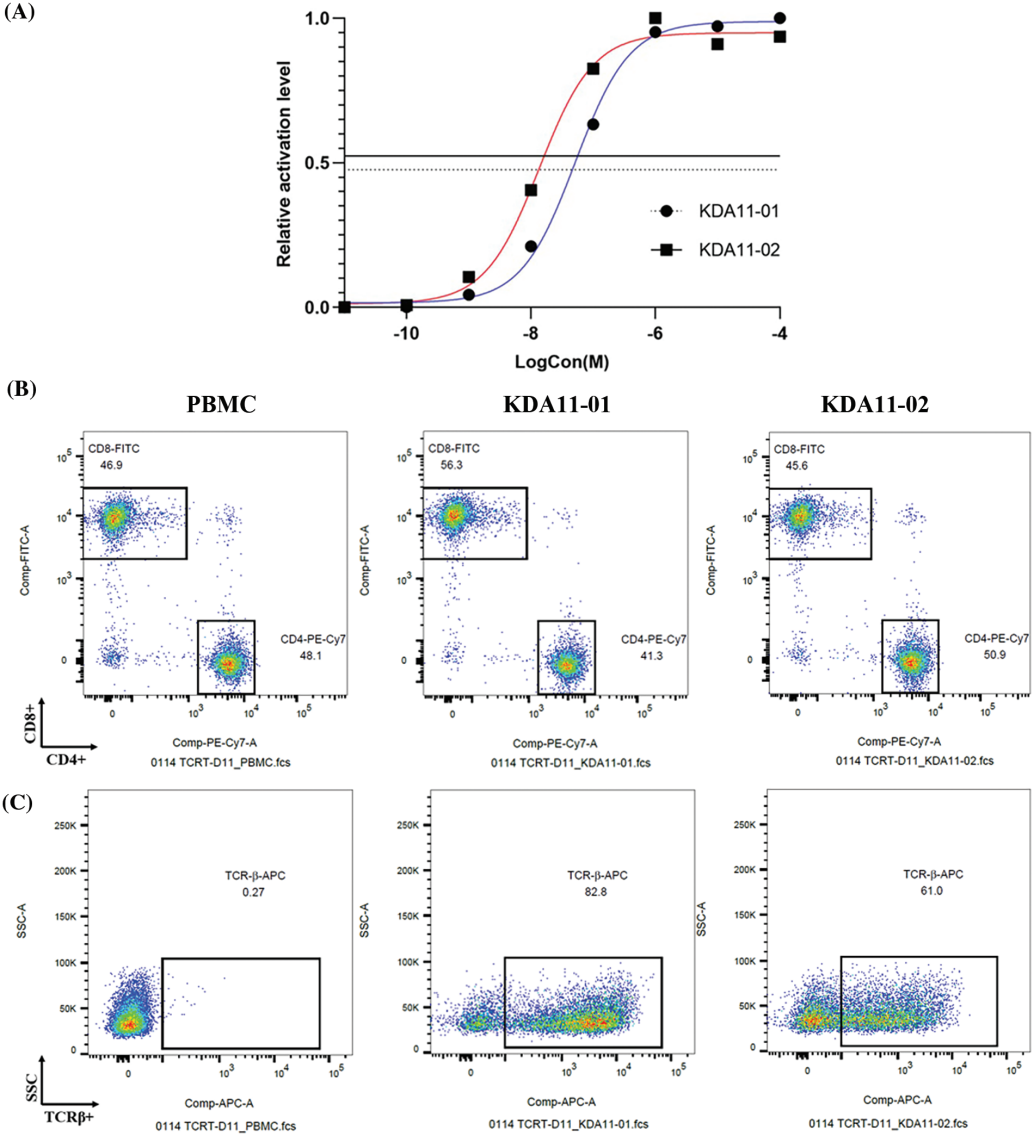
Evaluation of avidity and expression profiles of KDA11-01 and KDA11-02 TCRs. (A) shows the dose-response curves for KDA11-01 and KDA11-02 TCRs, illustrating their functional avidity. (B) and (C) display flow cytometry analyses of TCR expression in transduced T cells, highlighting CD4^+^ and CD8^+^ subsets, and TCR-β expression levels.

**Figure A1:**
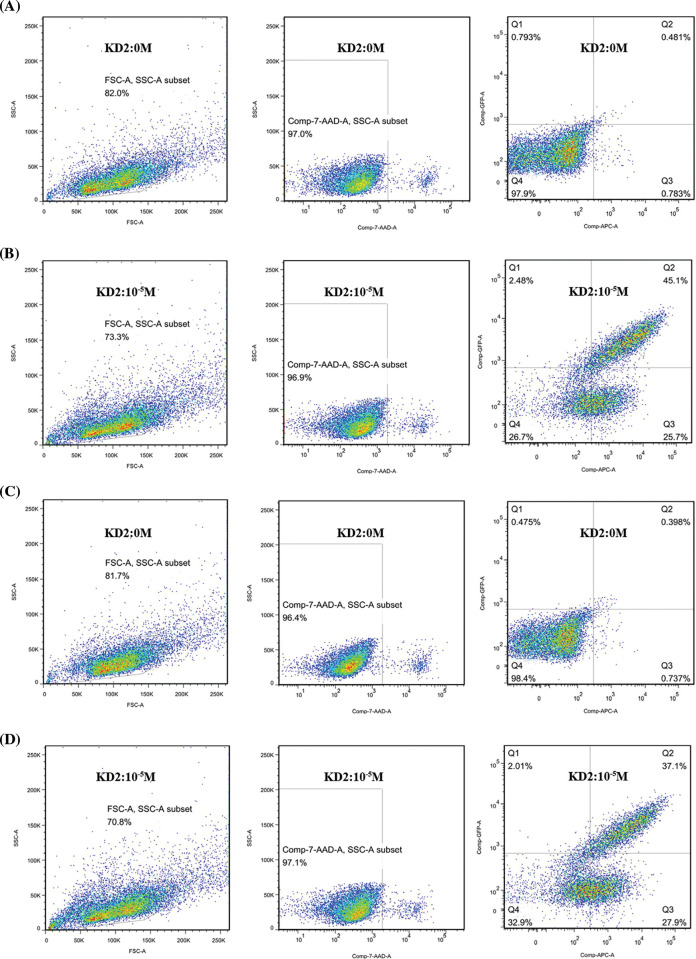
Activation of KDA11-01 and KDA11-02 TCR-Transduced Jurkat Cells. Flow cytometry analysis was used to assess the activation of TCR-transduced Jurkat reporter cells in response to specific antigens. (A) shows KDA11-01 TCR-transduced Jurkat reporter cells co-incubated with T2KO-A1101 cells without KD2 peptide loading, serving as a control to establish the gating strategy. (B) demonstrates the activation of KDA11-01 TCR-transduced Jurkat reporter cells following co-incubation with T2KO-A1101 cells loaded with 10^−5^ M KD2 peptide, indicated by the detection of reporter gene activation through flow cytometry. (C) shows KDA11-02 TCR-transduced Jurkat reporter cells co-incubated with T2KO-A1101 cells without KD2 peptide loading, confirming the gating strategy. (D) illustrates the activation of KDA11-02 TCR-transduced Jurkat reporter cells after co-incubation with T2KO-A1101 cells loaded with 10^−5^ M KD2 peptide, with reporter gene activation measured by flow cytometry.

**Figure A2:**
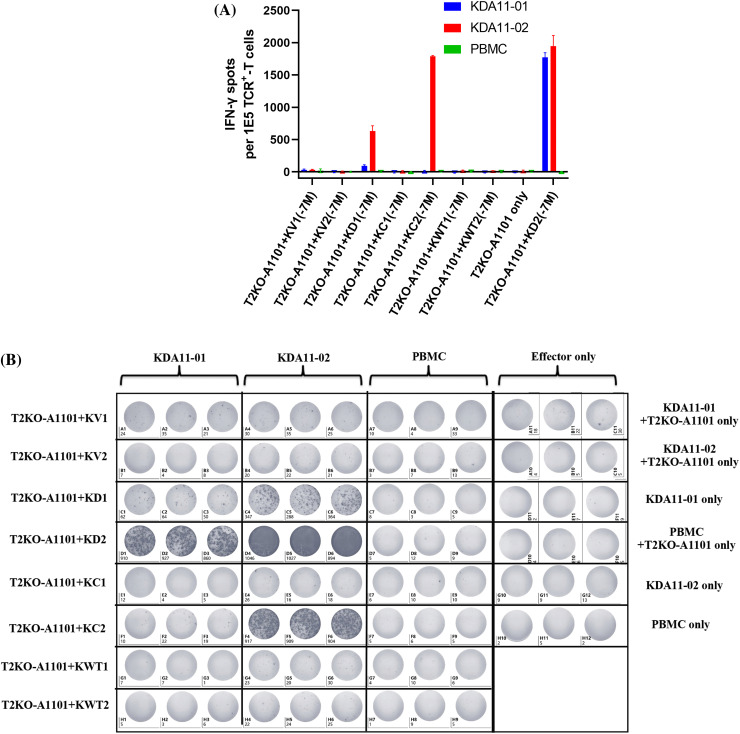
TCR-T homologous peptide analysis and ELISpot assay. This figure presents the results of the homologous peptide analysis and the ELISpot assay for KDA11-01 and KDA11-02 TCR-transduced T cells. Panel (A) shows the alignment of the KRAS G12D peptide with homologous peptides to assess potential cross-reactivity. Panel (B) depicts the ELISpot assay results, illustrating the frequency of IFN-γ-secreting T cells in response to stimulation with the KRAS G12D peptide and its homologs. This analysis was conducted to evaluate the specificity of the TCRs and ensure minimal cross-reactivity with similar peptides.

To evaluate the functional avidity and membrane stability of KDA11-01 and KDA11-02 TCRs, we assessed their antigen-peptide binding efficiencies (Fig. 3A). Jurkat reporter cells, transduced with these TCRs displayed potent reactivity to KD2 antigen peptides, with EC50 values calculated at 4.998 × 10^−8^ M for KDA11-01 and 1.333 × 10^−8^ M for KDA11-02. This indicates that both TCRs have high sensitivity to the KRAS G12D peptide, with KDA11-02 demonstrating slightly higher functional avidity. Further details regarding the functional avidity of KDA 11-01, KDA 11-02, and TK412 are provided in the Figs. A1 and A2.

Building upon previous research [26], which emphasizes the significance of T cell expression levels and membrane stability for optimal TCR function, we further examined these parameters in primary human T cells transduced with KDA11-01 and KDA11-02. Post-transduction, KDA11-01 exhibited a higher transduction efficiency in TCR-T cells compared to KDA11-02 (83.8% *vs*. 64.5%), suggesting that KDA11-01 has a superior membrane expression profile. Additionally, differences in CD4/CD8 expression ratios were observed between the two TCRs, with KDA11-01 showing a higher proportion of CD8^+^ T cells (Fig. 3B).

Further characterization of the transduced T cells was conducted using an anti-TCR-β antibody (Fig. 3C). The gating strategy for identifying KDA11-01 and KDA11-02 TCRs by flow cytometry is detailed in Fig. A1. The flow cytometry scatter plots revealed higher percentage of TCR-β expression in KDA11-01-transduced cells compared to KDA11-02. This observation aligns with differences in transduction efficiency and membrane stability. These findings highlight the effective expression and stability of the TCRs, which are critical for their potential therapeutic potential. Fig. A2 provides a detailed analysis of TCR-T cross-reactivity with homologous peptides, including peptide alignment and ELISpot assay results.

### Antigen-specific immunological activity and cytotoxicity of KDA11-01/02 TCR-transduced T cells

**Figure 4:**
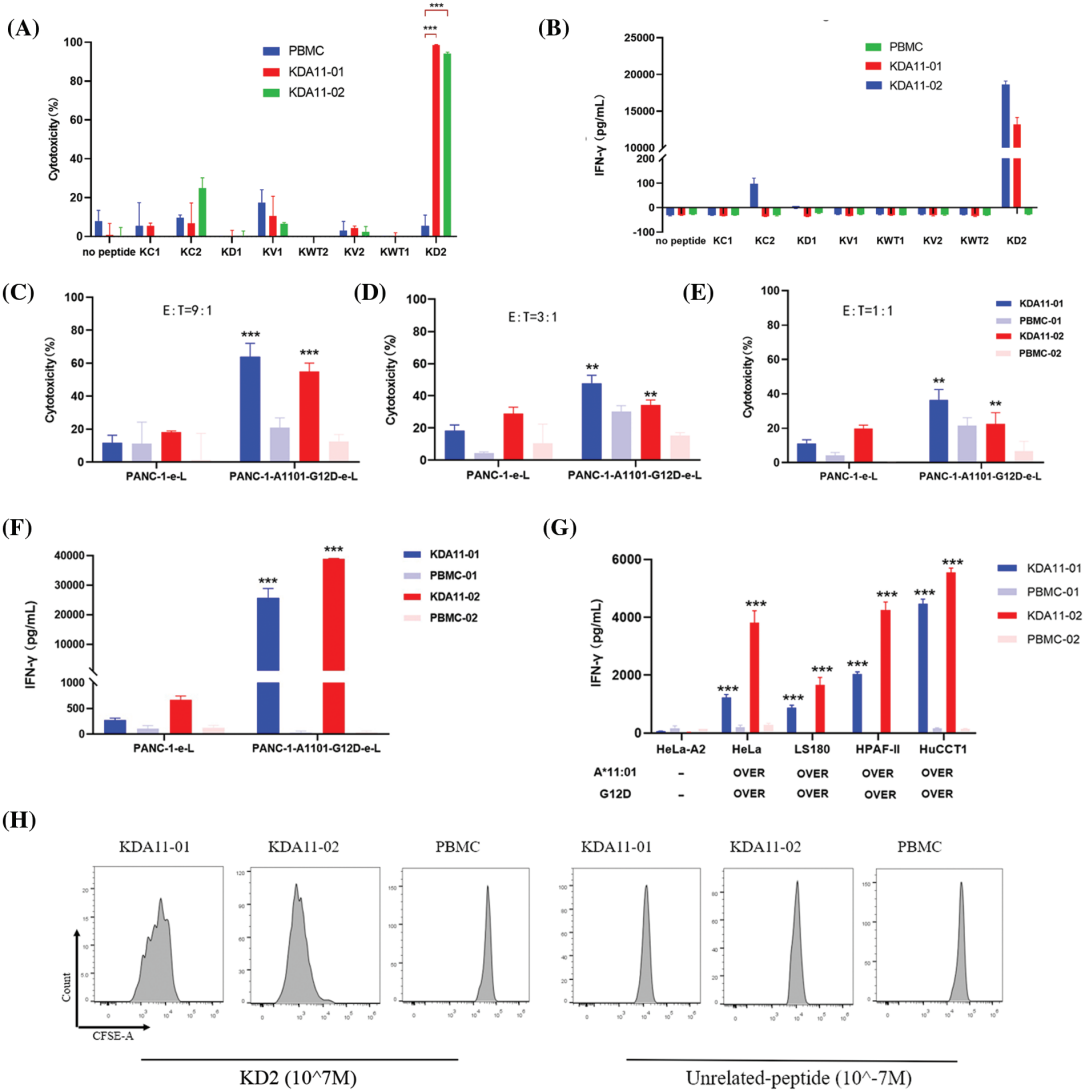
Function Analysis of KDA11-01 and KDA11-02 TCR-Transduced T Cells. (A) Cytotoxicity of KDA11-01 and KDA11-02 TCR-transduced T cells against T2KO-A1101 cells loaded with KD2 peptide and various control peptides, confirming TCR specificity. (B) IFN-γ secretion levels from TCR-transduced T cells co-cultured with T2KO-A1101 cells pulsed with different peptides, validating antigen specificity. (C–E) Cytotoxic efficacy against PANC-1 cells expressing HLA-A*11:01 and KRAS G12D at E ratios of 9:1 (C), 3:1 (D), and 1:1 (E). (F) IFN-γ secretion in co-cultures with PANC-1-A1101-G12D cells. (G) IFN-γ release by TCR-transduced T cells in co-culture with various HLA-A11:01 and KRAS G12D-expressing cancer cell lines, with HeLa-A2 as a negative control. (H) Proliferative response of TCR-transduced T cells after co-culture with T2KO-A1101 cells pulsed with KD2 or irrelevant peptides. **p < 0.01, ***p < 0.001.

The antigen-specific immunological activity and cytotoxic potential of KDA11-01/02 TCR-transduced T cells were assessed by coculturing them with T2KO-A1101 target cells that were pulsed with exogenous antigenic peptides, with non-transduced PBMCs serving as a control for effector cell activity. The TCR-transduced T cells displayed targeted cytotoxicity and IFN-γ production, with KDA11-01 TCR-transduced T cells showing particularly pronounced immune responses, evidenced by enhanced cytotoxicity and IFN-γ secretion upon exposure to peptide-pulsed T2KO-A1101 cells, in comparison to their KDA11-02 TCR-transduced counterparts (Fig. 4A,B). Intriguingly, while KDA11-02 TCR-transduced T cells induced substantial cytotoxicity in the presence of the KC2 peptide (Fig. 4A), the same cells did not produce a corresponding level of IFN-γ, as measured by ELISA when stimulated with KC2 (Fig. 4B). To resolve this observation, an ELISpot assay was employed with the identical peptide set including KC1, KC2, KD1, KD2, KWT1, KWT2, KV1, and KV2. However, this observation suggests that patient 2 (WFY210825) may also possess the KRAS G12C mutation. Due to the limitations inherent in using the Mabtech IRIS 2 ELISpot reader, particularly in cells D4 to D6 where spots overlapped, the actual spot count is likely higher than reported. These images are examples of the broader and more complex experimental outcomes encountered while optimizing and balancing the number of cells loaded and the ratio of effector to target cells. Further details and implications of these findings are discussed in the discussion section.

Meanwhile, KDA11-01/02 TCR-transduced T cells displayed negligible cytotoxicity and IFN-γ release in response to cells presenting non-antigenic or irrelevant peptides (KC1, KC2, KV1, KV2, KD1, KWT1, KWT2), confirming their antigen-specific immune responses (Fig. 4A,B). KDA11-01 demonstrated higher immunological activity than KDA11-02.

The activity of KDA11-01/02 was further tested against endogenously KRAS G12D-expressing tumor cells. The PANC-1 cell line was engineered to express both G12D and HLA-A11:01 (PANC-1-1101-G12D). These TCR-transduced T cells exhibited significant cytotoxicity and IFN-γ secretion against PANC-1-1101-G12D in a dose-dependent manner, with an enhanced response compared to PANC-1 cells without overexpression (Fig. 4C–F). Notably, KDA11-01 had a more pronounced cytotoxic effect than KDA11-02, whereas KDA11-02 elicited higher IFN-γ production (Fig. 4F). Additional experiments with HeLa, LS180, HPAF-II, and HuCCT1 cell lines, all overexpressing HLA-A*11:01 and KRAS G12D, revealed IFN-γ secretion by both TCRs, indicating potent antigen-specific immune activity (Fig. 4G).

Furthermore, we investigated T cell proliferation mediated by KDA11-01/02. T2KO-A1101 cells pulsed with KD2 peptides induced notable proliferation in KDA11-01 TCR-T cells, as evidenced by five distinct peaks, whereas unrelated peptides resulted in a single peak without proliferation (Fig. 4H). KDA11-02 TCR-T cells also increased, indicated by two distinct peaks at a lower fluorescence level. In contrast, the control PBMCs did not show proliferation with either KD2 or unrelated peptides (Fig. 4H). These findings suggest that KD2 peptides specifically stimulate the proliferation of KDA11-01/02 TCR-transduced T cells, underscoring their antigen specificity.

### Low alloreactivity of KDA11-01/02 against broad HLA repertoire

To evaluate the specificity of KDA11-01 and KDA11-02 TCRs for different HLA types, we generated a panel of 63 HLA cell lines derived from T2KO-LUC.2-TAP1 cells, which are HLA-deficient with restored TAP1 expression. This panel represents a broad range of the HLA class I variations found in the Chinese population. The goal of this experiment was to determine whether the KDA11-01 and KDA11-02 TCRs exhibit any unintended reactivity to non-target HLA types, thereby confirming their specificity for HLA-A*11:01 in the context of the KRAS G12D peptide.

**Figure 5:**
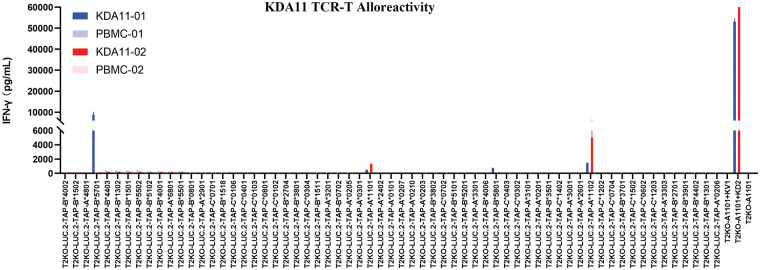
Specificity assessment of KDA11-01 and KDA11-02 TCRs against a broad panel of HLA class I variants.

Upon exposure to T2KO-A*11:01 cells pulsed with KD2 peptides, both KDA11-01/ and KDA11-02 TCR-transduced T cells robustly secreted IFN-γ, as expected. As shown in Fig. 5, these TCRs exhibited negligible responses to most of the tested HLAs. However, KDA11-01 showed some reactivity towards HLA B5701, B5801, and A1102 for KDA11-01 and HLA A1102, while KDA11-02 showed reactivity towards HLA-A*11:02. These findings indicate that both KDA11-01 and KDA11-02 TCRs exhibit minimal cross-activity with non-target HLA types, underscoring their potential for safe therapeutic application.

Fig. 5 illustrates the IFN-γ release from KDA11-01 and KDA11-02 TCR-transduced T cells upon exposure to 63 different HLA class I variant cell lines derived from T2KO-LUC.2-TAP1 cells, which are HLA-deficient with restored TAP1 expression. The results confirm the specificity of both TCRs for the HLA-A*11:01-restricted KRAS G12D_7-16_ epitope, showing minimal cross-reactivity with other non-target HLA types. Blue bars represent the response of KDA11-01, red bars for KDA11-02, and white bars indicate the control conditions.

## Discussion

In this study, we utilized the state-of-the-art single-cell TCR cloning technology to isolate and characterize two distinct TCRs, KDA11-01 and KDA11-02, specifically targeting the HLA-A*11:01-restricted KRAS G12D_7-16_ epitope. These TCRs, derived from two different patients with CRC and ICC, demonstrated high affinity, stable membrane integration, selective immunological activity, and cytotoxicity while maintaining a low alloreactivity profile [27,28].

Our refined method of amplifying TCRαβ chains with a 2A peptide sequence enhanced fusion efficiency and expression consistency, avoiding the need for mRNA and product purification. This innovation minimized template loss risks and streamlined the procedure [29–31]. Immunological assays confirmed that TCR-transduced T cells were selectively activated by T2KO-A11:01 cells presenting the KRAS G12D peptide, leading to significant IFN-γ production and targeted cytotoxicity [31,32].

However, we observed that KDA11-01 exhibited pronounced allo-reactivity towards HLA-B57:01, and both TCRs showed minor reactivity to HLA-A11:01 and A*11:02 alleles without neoepitope loading, suggesting potential cross-reactivity to ubiquitously expressed peptides presented by these HLA alleles. To address safety concerns, additional safety evaluations are necessary, including extended screenings against a broader range of HLA-presented peptide libraries and healthy tissue samples [33,34].

The selective activation of TCR-transduced T cells by T2KO-A11:01 cells presenting the KRAS G12D peptide, alongside the absence of reactivity to cells presenting wild-type or unrelated KRAS peptides, demonstrated the high specificity of KDA11-01 and KDA11-02. These TCRs also elicited strong immune responses against various tumor cell lines expressing both HLA-A11:01 and the KRAS G12D mutation, including PANC-1, HeLa, HPAF-II, and HuCCT1 [35–37].

The findings underscore the necessity of comprehensive safety assessments for patients harboring multiple KRAS mutations who might benefit from TCR-T therapy. Incorporating methodologies such as p-MHC tetramer binding, cell line recognition, and reactivity against extensive peptide libraries can enhance the safety profile of our TCR therapies [36,38].

Regarding the integration of murine sequences in TCR design, our approach included using mouse-derived constant regions (mTRAC, mTRBC) to enhance TCR pairing, crucial for stable expression on T cell surfaces. Additional disulfide bonds and hydrophobic mutations (LVL) in the transmembrane region of the mouse TCR alpha were introduced to prevent mismatches with human TCR components. This design strategy utilizes murine constant regions without reported off-target toxicity, a practice widely adopted due to its effectiveness in enhancing TCR pairing without significant immunogenic concerns, as detailed in the review by Shafer et al. [39].

In the context of ongoing clinical trials, the relevance of our study is highlighted by the Phase I/II study (ClinicalTrials.gov, Identifier: NCT05194735) that explores the efficacy of autologous T cells engineered to express TCRs reactive against neoantigens in patients with solid tumors. This study targets mutations like KRAS G12D in different cancer types, underscoring the clinical potential of our approach.

A noteworthy observation in our study was the differential functionality between KDA11-01 and KDA11-02, where KDA11-01 showed higher cytotoxicity, while KDA11-02 elicited greater IFN-γ secretion. This difference emphasizes the roles of TCR expression levels and antigen presentation in mediating effective anti-tumor responses [40–42]. The cytotoxic response by KDA11-02 to KC2 peptide further suggests potential recognition of additional KRAS mutation warranting further investigation [43].

This study, while demonstrating promising *in vitro* efficacy for KDA11-01 and KDA11-02, has several limitations. First, the safety of these TCRs requires comprehensive validation through *in vivo* studies, particularly to rule out off-target cross-reactivity and ensure their safety profile. Additionally, our current data is based on limited patient samples and specific HLA types, which may not fully represent the broad patient population. Therefore, further research involving a more extensive and diverse cohort is essential. Additionally, although murine sequences were incorporated to enhance TCR pairing, the long-term immunogenicity of these sequences needs to be thoroughly investigated. Finally, while the study highlights the potential for clinical application, rigorous *in vivo* studies and extensive safety screenings against a wide array of human tissues are crucial before considering clinical trials.

This research significantly advances TCR-based treatments for cancers with KRAS G12D mutations, offering potential new therapeutic options for patients [39,44,45]. Our systematic approach to cloning human TCRs highlights the promising *in vitro* effectiveness of KDA11-01 and KDA11-02. However, the transition to clinical application requires further *in vivo* validation to establish their therapeutic potential and safety.

## Conclusions

This study presents a robust and efficient methodology for the isolation and cloning of human TCRs, using single-cell TCR gene amplification, followed by cloning into expression vectors. The engineered TCRs, KDA11-01 and KDA11-02, transduced into T cells, demonstrated significant *in vitro* efficacy in targeting and eliminating malignant tumor cell lines harboring the KRAS G12D mutation within the context of HLA-A*11:01. These TCRs not only demonstrated their specificity and potency in eliciting anti-tumor responses but also maintained low alloreactivity and exhibited no off-target effects, which underscores their therapeutic potential. While the *in vitro* success of these TCRs is promising, extensive *in vivo* studies are crucial to further validate their efficacy and safety comprehensively before processing to clinical trials targeting oncogenic mutations.

## Data Availability

Supplementary data will be available online.
